# Using Linked Data to Explore the Relationship Between Walking-Friendliness of Neighbourhoods, Physical Activity and Body Mass

**DOI:** 10.23889/ijpds.v1i1.343

**Published:** 2017-04-19

**Authors:** Nancy Ross, Kaberi Dasgupta, Claudia Sanmartin, Rania Wasfi, Samantha Hajna, Sarah Mah

**Affiliations:** 1 McGill University; 2 Statistics Canada

## Objectives

To demonstrate the methodology and results for linking measures of neighbourhood walking-friendliness or "walkability" to Canadian health surveys and Canadian health surveys linked to administrative health care records. 

## Approach

We linked multiple measures of neighbourhood walkability to hundreds of thousands of 6-digit postal codes of respondents to three large Canadian surveys using geographic information systems and anonymized banks of postal codes.

## Results

Long term exposure to walkable neighbourhoods was associated with increased reporting of walking for utilitarian purposes. Moving to a high walkable neighbourhood from a low walkable neighbourhood was associated with a full unit decrease in the body mass index of Canadian men over a 12-year follow-up. In a linkage of walkability measures to respondents who wore biosensors for a one-week period in several Canadian cities, we found that neighbourhood walkability was associated with increased reporting of utilitarian walking but not overall physical activity and step counts as measured by biosensors.

## Conclusion

There is potential for walkable neighbourhoods to influence physical activity and body weight of Canadians which is more evident when individuals are followed for long periods of time.

